# Fertility in the Shadow of Cancer: Experiences of Reproductive Loss Among Women with Gynecological Cancers in Ghana

**DOI:** 10.1371/journal.pone.0343859

**Published:** 2026-08-14

**Authors:** Agani Afaya, Donald Babamomba Amenah, Fahd Chambas, Promise Aidoo, Ohene Addo Gideon, Mavis Vidzor, Bernice Aidoo, Richard Adongo Afaya, Mabel Apaanye Avane, Silas Selorm Daniels-Donkor, Dennis Bomansang Daliri, Solomon Mohammed Salia

**Affiliations:** 1 Department of Nursing, School of Nursing and Midwifery, University of Health and Allied Sciences, Ho, Ghana; 2 Department of Family and Community Health, University of Maryland School of Nursing, Baltimore, Maryland, United States of America; 3 Department of Midwifery and Women’s Health, School of Nursing and Midwifery, University for Development Studies, Tamale, Ghana; 4 School of Nursing, University of Virginia, Charlottesville, Virginia, United States of America; 5 Presbyterian Psychiatric Hospital, Bolgatanga, Ghana; Universidad Peruana Cayetano Heredia Facultad de Medicina, PERU

## Abstract

**Background:**

Gynecological cancers remain a threat to women’s health in Ghana, where the burden is shaped by late diagnosis, limited specialized care, and treatment-related infertility. Surgery, chemotherapy, and radiotherapy can damage the ovaries and uterus, causing premature ovarian failure, early menopause, and loss of reproductive potential. In a pronatalist context where motherhood supports identity, marriage stability, and social standing, infertility carries a psychosocial burden. Yet, the lived experiences of women with gynecological cancer-related infertility remain poorly understood.

**Objective:**

This study explored the experiences of reproductive loss among women with gynecological cancers receiving care at a tertiary referral hospital in Ghana.

**Methods:**

A qualitative descriptive design guided the study. Fourteen women aged 22–48 years with cervical, ovarian, uterine, or endometrial cancers, who had completed or were undergoing treatment, were purposively recruited. Semi-structured interviews (30–45 minutes) were conducted face-to-face or by telephone in private settings in English, Twi, or Ewe by trained nurses, audio-recorded, transcribed verbatim, and analyzed inductively using thematic analysis.

**Results:**

Three interconnected themes and thirteen subthemes emerged from the data: (1) psychological and emotional experiences, (2) social challenges, and (3) coping and resilience strategies. Fertility loss was experienced as a threat to womanhood, motherhood, and relational security, especially among younger, single, and childless participants. Women undergoing active treatment emphasized symptom burden, whereas those post-treatment focused on childbearing loss. Participants described incompleteness, fear of rejection, anxiety, denial, and shattered aspirations, while social stigma and strained intimate relationships intensified distress. These experiences were compounded by treatment-related symptoms, financial strain, and interrupted education. Coping drew on adherence to treatment, dietary changes, prayer, and family support, which helped sustain hope and resilience.

**Conclusion:**

Reproductive loss among women with gynecological cancers in Ghana is a psychosocial, sociocultural, and economic burden that affects quality of life. Holistic, culturally sensitive care should integrate fertility counseling, psychosocial support, symptom management, financial protection, and guidance on alternative family-building options such as adoption.

## Introduction

Gynecological cancers remain a major yet often under-recognized threat to women’s health worldwide, with profound consequences for survival, quality of life, and health systems, particularly in low- and middle-income settings such as Ghana [1]. In 2022, approximately 1.47 million women were newly diagnosed with gynecological cancers, and an estimated 680,000 women died from these diseases globally, representing more than 15% of all cancer diagnoses and cancer-related deaths among women [2,3]. This burden is projected to rise substantially by 2050, with the sharpest increases expected in low- and middle-income regions, especially sub-Saharan Africa [1]. In Ghana, gynecological cancers, particularly cervical and ovarian cancers, constitute a major source of cancer-related morbidity and mortality. Cervical cancer alone accounts for an estimated 2,797 new cases and 1,699 deaths annually, corresponding to age-standardized incidence and mortality rates of approximately 27.0 and 16.9 per 100,000 women, respectively [4,5]. These outcomes are frequently shaped by late-stage diagnosis, low screening uptake—estimated at about 7% among eligible women—and constrained access to specialized oncology services, resulting in substantial out-of-pocket expenses and poorer survival outcomes [6,7].

Advances in oncological management, including chemotherapy, surgery, pelvic radiotherapy, and hormone therapy, have improved survival among women with gynecological cancers. However, these treatments frequently carry gonadotoxic effects, including premature ovarian failure, early menopause, and direct damage to reproductive organs, resulting in cancer-related infertility [4,8–10]. As survival improves and the number of reproductive-aged cancer survivors increases, oncofertility has emerged as a critical intersection between oncology and reproductive medicine. Oncofertility encompasses the clinical and research efforts to preserve and restore reproductive potential in cancer patients, including fertility-sparing surgical approaches, ovarian stimulation and oocyte or embryo cryopreservation, and ovarian tissue preservation [11,12]. Although such interventions are increasingly standard in high-income countries, access to fertility preservation services in low- and middle-income countries, including Ghana, remains highly constrained. These limitations are driven by inadequate infrastructure, high costs, the absence national guidelines, and poorly integrated cancer–reproductive care pathways [1,11,13]. A recent multicenter study in Ghana found that fertility preservation awareness among women with breast cancer was only 39.3%, with cost cited as the primary barrier (73.5%), underscoring the extent of the unmet need [8]. This gap is especially consequential for women with gynecological cancers, where the reproductive organs are directly affected, and fertility loss is not merely a treatment side effect but frequently an inevitable clinical outcome.

The implications of cancer-related infertility extend well beyond biology and represent a profound psychosocial burden for women of reproductive age. In Ghana, motherhood is deeply intertwined with women’s social identity, marital stability, and community standing [14,15]. Childbearing is widely regarded as a marker of womanhood, a foundation of marriage, and a condition for social and lineage continuity, values that are pervasive across ethnic groups in Ghana [14,15]. In this pronatalist social context, involuntary childlessness carries intense stigma, with infertile women risking marital dissolution, social marginalization, and loss of community respect [14,16]. As a result, cancer-related infertility is experienced not only as a clinical consequence of treatment but also as a socially meaningful loss that may undermine identity, relational worth, and perceived future belonging. Psychosocially, cancer-related infertility is associated with a spectrum of mental health consequences, including depression, anxiety, grief, identity disruption, and decisional regret [17,18]. The uncertainty surrounding fertility loss can also create a sense of bodily incompleteness and strain intimate relationships [18,19]. Among women with gynecological cancers, psychological distress related to fertility and pregnancy is a documented survivorship burden, with studies showing that depression and anxiety disorders independently increase mortality risk in this population [20,21]. These findings underscore the necessity of integrating psychosocial and fertility-specific support into cancer care, particularly in resource-constrained settings where such services are absent.

Despite growing global attention to oncofertility and cancer-related infertility, critical service and evidence gaps persist in Ghana. Fertility counseling is rarely embedded within gynecological oncology care in most public facilities; fertility preservation services are largely unavailable outside a few private clinics in Accra; and formal cancer–reproductive care pathways are not established within the public health system [8,13]. As a result, women receiving cancer care at tertiary public hospitals, including Ho Teaching Hospital, which serves the Volta and Oti Regions, often undergo fertility-compromising treatment without structured pre-treatment counseling, fertility-sparing alternatives, or post-treatment psychosocial and reproductive support. These constraints are further compounded by high out-of-pocket costs, which place the limited available fertility services beyond the reach of most women [6,8].

Existing qualitative evidence on cancer-related infertility in Ghana and sub-Saharan Africa remains sparse and tends to focus on biomedical or epidemiological associations rather than the lived, culturally situated experiences of women navigating fertility loss in the context of cancer [22]. Specifically, it remains unknown how Ghanaian women with gynecological cancers emotionally experience and interpret reproductive loss; how they make treatment and reproductive decisions under conditions of limited information and absent fertility preservation options; what coping strategies they draw upon; and how cultural constructions of womanhood and motherhood shape their experience of fertility loss and survivorship. These questions are not merely academic: they define the content and form of the psychosocial and fertility-sensitive care that women need and are not receiving.

Accordingly, this study explored the experiences of reproductive loss among women with gynecological cancers receiving care at Ho Teaching Hospital in the Volta Region of Ghana, with particular attention to the emotional, social, cultural, and coping dimensions of their experiences.

## Methods

### Study design

This study employed a qualitative descriptive design to explore the experiences of reproductive loss among women with gynecological cancers. Qualitative description was chosen because it is well-suited to generating rich, direct accounts of experience from participants’ own perspectives, particularly for sensitive health topics where in-depth understanding is sought rather than hypothesis testing or theoretical interpretation [23–25]. The study is epistemologically grounded in constructivism, which holds that knowledge and meaning are socially and experientially constructed and that participants’ accounts of illness and reproductive loss are shaped by personal biography, cultural context, and relational positioning [25]. This epistemological stance informed the interpretive orientation of both data collection and analysis. The study was conducted and reported in accordance with the Consolidated Criteria for Reporting Qualitative Research (COREQ) [26].

### Study Setting

The study was conducted at the Ho Teaching Hospital (HTH), a public tertiary referral facility in Ho, the capital of the Volta Region of Ghana. HTH serves as the regional referral center for the Volta and Oti Regions, receiving complex and late-stage cases from district and municipal hospitals across a broad geographic catchment area. As a national teaching hospital affiliated with the University of Health and Allied Sciences (UHAS), HTH provides specialized multidisciplinary care across numerous clinical disciplines. Participants were recruited specifically from the gynecology outpatient clinic and the chemotherapy day unit, where women with gynecological cancers receive ongoing treatment and follow-up. Care at HTH is provided across both outpatient and inpatient settings. Gynecological oncology services include surgical management, chemotherapy, and palliative care. It should be noted that at the time of the study, HTH had limited fertility preservation services, and limited formally structured fertility counseling pathway integrated into its gynecological oncology care. The Volta Region is characterized by ethnic and linguistic diversity, with the Ewe language predominant; cultural norms around fertility, marriage, and motherhood are deeply pronatalist, and childbearing carries strong social and identity significance for women in this region [14,15].

### Study population

The study population consisted of women of reproductive age who had received a diagnosis of gynecological cancer and were receiving or had received treatment at HTH. Given the qualitative and experience-focused nature of the study, the inclusion and exclusion criteria were designed to ensure that participants had sufficient direct experience of cancer diagnosis and treatment-related fertility impacts to speak meaningfully to the study aims.

### Inclusion criteria

The inclusion criteria for participants in this study were as follows:

- Women aged 18–49 years at the time of data collection.
- Women with a confirmed histological or clinical diagnosis of any gynecological cancer type, including cervical, ovarian, uterine, endometrial, vaginal, or vulvar cancers.
- Women who have completed or are currently undergoing treatment (surgery, chemotherapy, and/or radiotherapy).
- Women who were willing and able to participate and who provided written informed consent.

### Exclusion criteria

The exclusion criteria involved:

- Women diagnosed with non-gynecological cancers.
- Women with a known diagnosis of infertility or documented reproductive dysfunction pre-dating their cancer diagnosis
- Women whose current clinical condition (e.g., critical illness) would preclude safe, meaningful participation in a research interview.
- Women with a clinically documented psychiatric disorder of sufficient severity to impair capacity to provide meaningful, reflective responses.
- Women who were newly diagnosed but had not yet commenced any treatment.

### Sampling strategy

Purposive sampling, specifically criterion-based purposive sampling, was employed to identify and recruit participants who met the inclusion criteria and had direct, firsthand experience relevant to the study objectives. Purposive sampling was selected because it allows for the intentional identification of information-rich participants who can provide in-depth and meaningful insights into the phenomenon under investigation [27]. To ensure depth and variation in experiences, sampling was guided by attention to variation across key characteristics, including age group, cancer type (cervical, ovarian, endometrial, or uterine), treatment modality (surgery, chemotherapy, radiotherapy, or combination), marital status, and parity.

### Data collection method and procedure

Data were collected from 1st November 2025 to 14th November 2025 through individual semi-structured interviews. A semi-structured interview guide was developed based on a systematic review of the relevant literature [28] and aligned with the study’s objectives. The guide was reviewed for cultural appropriateness and clinical relevance by two independent experts (a gynecologic oncology nurse and a qualitative health researcher) before finalization and was pre-tested with three women of similar characteristics recruited outside the main study sample. Minor revisions were made to the guide, and participants who participated in the pre-test were not included in the main analysis. The guide covered five domains: background and sociodemographic information; understanding and perceptions of gynecological cancer and fertility; physical, emotional, and psychological experiences; social and cultural challenges; and coping strategies. It included both open-ended questions and probing prompts to encourage participants to elaborate on their responses and share in-depth experiences.

Eligible individuals were contacted, informed about the purpose of the study, and provided with participant information sheets. Written informed consent was obtained before participation. No enrolled participant withdrew from the study. Interviews were conducted by two bachelor’s-prepared registered nurses who were trained in qualitative interviewing, had prior experience in qualitative health research, and had no clinical or supervisory relationship with any participant. Both interviewers were female, a choice intended to facilitate participant comfort given the sensitive, gendered nature of the topic. Interviewers maintained a reflexive journal throughout data collection to document observations, emerging analytic impressions, and reflections on potential researcher influence on the data. Interviews were primarily conducted face-to-face in private consultation rooms within the gynecology outpatient clinic and chemotherapy unit, ensuring confidentiality and a safe environment for disclosure. For participants (n = 4) unable to attend in person due to distance, health status, and logistical constraints, telephone interviews were conducted as an alternative. To address the potential for differences in data richness between face-to-face and telephone modalities, telephone interviewers used additional probing and verification prompts to compensate for the absence of non-verbal cues, and reflexive notes documented any apparent differences in depth. Overall, no substantive thematic differences were observed between face-to-face and telephone data.

Interviews were conducted in English, Twi, or Ewe, according to the participant’s preference and language of fluency. Interviews in Twi or Ewe were conducted by a bilingual team member, translated into English, and verified by a second bilingual team member; any discrepancies were resolved through discussion to protect meaning fidelity. All sessions were audio-recorded with participants’ written permission and lasted between 30 and 45 minutes. Audio recordings were transcribed verbatim by a team member who was not present at the interview, providing a degree of analytical distance during transcription. Transcripts were stored in password-protected files on an encrypted institutional device accessible only to the research team. Participant identifiers were replaced with anonymous codes (Woman A through Woman N) across all transcripts, records, and reports. Data collection and preliminary analysis occurred concurrently, with recruitment continuing until thematic saturation was confirmed. Saturation was determined to be reached when successive interviews produced no additional codes and themes were robustly supported by data from participants [29]. Saturation was achieved after 14 interviews.

### Data analysis

Thematic analysis was employed using an inductive approach following the six-phase framework of Braun and Clarke [30]: (1) familiarization with the data, (2) generating initial codes, (3) searching for themes, (4) reviewing themes, (5) defining and naming themes, and (6) producing the report. Inductive thematic analysis was appropriate here because it allows meaning and patterns to emerge from the data itself, without imposing a predetermined theoretical framework, thereby preserving fidelity to participants’ situated and culturally embedded accounts of reproductive loss.

Analysis proceeded iteratively and concurrently with data collection. After each interview, verbatim transcripts were read in full by at least two members of the research team to achieve data immersion. All transcripts were coded independently to minimize individual interpretive bias. Initial codes, capturing all meaningful data segments relevant to the research questions, were generated line by line. These were compared across transcripts and organized into candidate themes based on patterns of shared meaning. Candidate themes were reviewed iteratively for internal coherence, distinctiveness, and grounding in the data; some were modified, merged, or subdivided into subthemes during this stage. Discrepancies between analysts in code assignment or theme interpretation were resolved through structured discussion until consensus was reached, a process documented in a shared audit trail. Thematic saturation influenced analytical decisions throughout this process: when successive interviews no longer generated new codes, the analytical team reviewed the code set for completeness and determined that saturation had been reached, confirming the adequacy of the sample for the study’s analytical aims.

Member checking was incorporated as an analytic integrity strategy: at the close of each interview, participants were invited to review and verify the researcher’s summary of key points discussed. Following preliminary thematic development, a subset of participants was also contacted to review summary findings for accuracy and resonance with their experience. Themes were refined in response to this feedback. The final analytical narrative was constructed to represent the breadth and depth of participants’ experiences, supported by direct quotations attributed by anonymous participant codes to maintain transparency about evidential grounding.

### Rigor

Trustworthiness was ensured through systematic, operationalized strategies across four quality criteria: credibility, dependability, confirmability, and transferability [31]. Credibility was strengthened through multiple specific mechanisms. Member checking was conducted at two points: during each interview, participants were invited to clarify or expand on their accounts, and summary findings were shared with a subset of six participants post-analysis for verification. Prolonged engagement, in the context of this study, was operationalized not as extended fieldwork duration but as deep, iterative immersion in the transcripts through multiple rounds of reading and re-reading prior to and during coding—a form of analytic engagement appropriate to interview-based qualitative description (25). Reflexive memos were maintained by both interviewers throughout data collection and analysis, documenting assumptions, potential biases arising from the research team’s professional backgrounds in nursing and public health, and analytic decisions, thereby supporting ongoing awareness of researcher influence on interpretation.

Dependability was achieved by maintaining a comprehensive and transparent audit trail, which documented all methodological decisions from study design through to final theme definition, including rationale for sampling decisions, interview protocol adjustments, coding revisions, and theme development. This trail was maintained jointly by two members of the analytical team. Confirmability was supported through investigator triangulation: all 14 transcripts were independently coded by two analysts, and coding outputs were compared systematically.

Divergences in code assignment or thematic interpretation were recorded in the audit trail and resolved through consensus discussion, rather than by deferring to one analyst’s judgment. Data triangulation was also achieved through the concurrent analysis of interview data and reflexive field notes, which documented contextual observations during face-to-face interviews and helped identify nonverbal dimensions of participants’ experiences. Peer debriefing was conducted with two colleagues external to the study team (one with expertise in qualitative oncology research and one in psychosocial reproductive health), who reviewed the codebook and candidate themes at two stages of analysis. Their questions and critical observations were documented and used to refine thematic definitions. Transferability was enhanced through thick, contextualized descriptions of the study setting (HTH, Volta Region), participant characteristics, the absence of fertility preservation services, local cultural norms surrounding childbearing, and the broader sociocultural context of care, facilitating consideration of the findings in similar Ghanaian and sub-Saharan African contexts.

### Ethical considerations

Ethical approval for this study was obtained from the University of Health and Allied Sciences Research Ethics Committee (Approval No. UR199/1125; validity: October 2025–October 2026). In addition, institutional permission to conduct the study was granted by the administration of Ho Teaching Hospital, including the Head of the Department of Obstetrics and Gynecology. All research activities were conducted in accordance with the principles of the Declaration of Helsinki and established ethical standards of respect for persons, beneficence, non-maleficence, justice, and confidentiality [32].

Given the sensitivity of discussions surrounding fertility loss, reproductive identity, and intimate relationships, particular attention was paid to psychological safety during recruitment and data collection. No financial compensation or transportation reimbursement was provided because interviews were scheduled alongside participants’ routine clinic visits, minimizing additional travel and logistical burden. The research team and ethics committee determined that participation did not impose substantial additional costs or inconvenience warranting reimbursement.

Written informed consent was obtained from all participants prior to data collection. Consent forms and participant information sheets were available in English, Twi, and Ewe to ensure that participants fully comprehended the study’s purpose, procedures, risks, benefits, and voluntary nature. All participants were literate and able to provide written consent. Participants were explicitly informed of their unrestricted right to withdraw at any stage without effect on their clinical care.

Confidentiality was rigorously protected. All participants were assigned anonymous codes (Woman A through to Woman N) in place of names across all transcripts, notes, and reports. Audio recordings and transcripts were stored in password-protected, encrypted files accessible only to named members of the research team. For telephone interviews, a private and secure telephone line was used, and participants were asked to confirm at the start of each call that they were in a private location where their responses could not be overheard, to ensure confidentiality equivalent to in-person interviews. All participants were monitored continuously for signs of emotional distress. Where participants became visibly emotional, interviews were paused; participants were reminded of their right to discontinue, and reassurance and support were provided. Where appropriate, participants were referred to HTH’s psychosocial support services for additional assistance. The sensitive nature of the topic was recognized throughout, and interviewers were briefed on trauma-informed communication practices prior to data collection.

## Results

### Sociodemographic characteristics of participants

The study recruited 14 participants to reach saturation. Participants’ ages ranged from 22 to 48 years, with most aged 20–29 years (6). The majority were single (9) and had no children (9). Cervical cancer was the most common diagnosis (7), followed by ovarian cancer (5), while uterine and endometrial cancers accounted for one case each. In terms of occupation, teachers and traders were the most represented groups (3 each), followed by students (3), unemployed participants (2), and one participant each who was a manager, farmer, and orderly (1 each). Nearly half had attained tertiary education (6). Most participants were of Ewe ethnicity (10), and all identified as Christians (Table 1).

**Table 1:** Socio-demographic information of participants.

Participants	Age (Years)	Marital status	Number of children	Type of cancer	Educational level	Occupation	Religion	Ethnicity
Woman A	29	Single	None	Ovarian	Tertiary	Manager	Christian	Ewe
Woman B	22	Single	None	Cervical	SHS	Student	Christian	Ewe
Woman C	27	Married	Four	Cervical	Primary	Farmer	Christian	Ewe
Woman D	47	Single	None	Uterine	Tertiary	Teacher	Christian	Ewe
Woman E	40	Single	None	Ovarian	Tertiary	Teacher	Christian	Ewe
Woman F	38	Single	None	Cervical	SHS	Trader	Christian	Ewe
Woman G	48	Married	Four	Endometrial	Primary	Unemployed	Christian	Ewe
Woman H	38	Married	Two	Ovarian	SHS	Unemployed	Christian	Akan
Woman I	29	Single	None	Cervical	Primary	Trader	Christian	Ewe
Woman J	27	Single	None	Ovarian	JHS	Trader	Christian	Akan
Woman K	31	Married	One	Cervical	SHS	Orderly	Christian	Ewe
Woman L	35	Married	Three	Ovarian	Tertiary	Teacher	Christian	Ewe
Woman M	30	Single	None	Cervical	Tertiary	Student	Christian	Akan
Woman N	23	Single	None	Cervical	Tertiary	Student	Christian	Akan

### Presentation of themes

The themes were developed from patterns identified in participants’ narratives, reflecting the psychological, emotional, social, and cultural dimensions of living with the condition. Each theme is accompanied by subthemes that provide a deeper understanding of the specific aspects within these broader categories. In all, the analysis yielded three themes and thirteen subthemes. Table 2 outlines the major themes and corresponding subthemes identified in the study.

**Table 2:** Presentation of emerged themes and sub-themes.

Themes	Subthemes
**Theme 1:** Psychological and emotional experiences	1. Sense of incompleteness 2. Fear of rejection 3. Anxiousness 4. Terrifying moments 5. Denial of diagnosis 6. Stressful experiences 7. Shattered aspirations
**Theme 2:** Social challenges	1. Stigmatization 2. Impaired sexual and intimate relationships
**Theme 3:** Coping and resilience strategies	1. Adherence to medication 2. Dietary and lifestyle modifications 3. Faith-based acts (praying) 4. Moral encouragement

### Theme 1: Psychological and emotional experiences

Most participants faced a range of psychological challenges, including feelings of incompleteness and fear of rejection. The experiences of participants extended beyond the psychological domain into deep emotional involvement, with most reporting feelings of fear, anxiety, denial, stress, and brokenness following their diagnosis. The narratives revealed how gynecological cancer profoundly affected their emotional well-being, shaping their perceptions of life, health, and motherhood.

#### Subtheme 1: Sense of incompleteness.

A dominant emotional thread expressed by participants was a profound sense of incompleteness stemming from the reality of infertility. In the Ghanaian cultural context, womanhood is deeply intertwined with motherhood, and the ability to conceive is often seen as a core marker of feminine identity and social worth. For many women, the confirmation of infertility triggered feelings of personal inadequacy, social failure, and internal conflict. Participants described a loss that extended beyond biological motherhood by saying:

*Sometimes I feel incomplete as a woman because of the fear and thought that I may not be able to give birth.* ***Woman A***

*At first, I felt incomplete as a woman because I could no longer have children. I used to think that being able to give birth was what made a woman whole, but through this experience, I have learned that womanhood is more than that. It is about strength, care, and love. I now believe that motherhood is not only about giving birth but also about nurturing and caring for others. My view of life and womanhood has changed; I have become stronger and more understanding, and I now value emotional and spiritual strength more than physical ability.* ***Woman F***

#### Subtheme 2: Fear of rejection.

Participants frequently expressed a deep-seated fear of rejection, which significantly shaped their decisions regarding romantic relationships. The diagnosis of infertility created anxiety over potential social stigma and partner abandonment, leading many to avoid intimate relationships altogether. Participants described how their diagnosis reshaped their social lives, prompting them to withdraw from pursuing romantic involvement to avoid anticipated judgment or emotional pain. They expressed this concern by saying:

*I was not in a romantic relationship before my diagnosis, and after knowing my condition, I have lost interest in entering into one because I am afraid of being rejected due to infertility. I haven’t told anyone else apart from them because I fear being stigmatized or pitied.* ***Woman F***

*I didn’t have a partner; I don’t intend having one again. I can’t conceive, and most of our men want women who can give them children. […] I need to focus on my education.* ***Woman N***

*Since I could no longer conceive, my husband started becoming distant, and I constantly feared that my condition would destroy our marriage. Sometimes, I felt like I was failing as a wife because I could not give him more children*. ***Woman K***

#### Subtheme 3: Anxiousness.

The uncertainty surrounding prognosis, treatment, and fertility outcomes generated high levels of anxiety among participants. Many reported that the emotional stress of their diagnosis affected both personal decisions and social interactions. They expressed their worries by saying:

*I was sad and anxious about the future since I knew I wouldn’t be able to conceive anymore. It didn’t affect my relationship with my family (mum and dad), but they were rather supportive and encouraged me to be positive. I was not in a romantic relationship prior to the diagnosis. I do feel it has negatively influenced my decision not to enter into a romantic relationship because of my infertility.* ***Woman F***

*I was stressed and worried about my health and life when I was first told about my condition. I was informed that the surgery would make me unable to give birth, but I wasn’t bothered since I already had four children.* ***Woman G***

*Emotionally, I was stressed and anxious, and I wasn’t stigmatized because nobody knew about my condition…. I decided to keep my condition to myself because I was afraid of how people would react if they found out I could no longer have children. Sometimes, staying silent felt easier than facing people’s questions and judgment.* ***Woman E***

#### Subtheme 4: Terrifying moments.

Participants described being terrified at the moment of diagnosis, often fearing death, the unknown progression of the disease, and social judgment. Many emphasized how confusing and overwhelming the situation felt while trying to stay strong in the face of fear, stating:

*I went to the hospital, and after several tests, the doctor said there was a tumor on my ovary. I was very scared at first, but I tried to stay strong.* ***Woman B***

*I was very scared and confused because I didn’t know much about it. The treatment journey was difficult, with constant checkups and fear of what would happen next.* ***Woman E***

*I was afraid of dying and also of what people might say. However, with time, my parents encouraged me and supported me through the treatment. The surgery was painful, but I thank God that I survived it. Living with the condition has taught me to be strong and appreciate life more.* ***Woman F***

#### Subtheme 5: Denial of diagnosis.

Participants reported that after the diagnosis, they initially refused to believe it, struggling to accept that they had cancer. The disbelief was often coupled with worry and emotional turmoil, making it difficult to process the news. They expressed their disbelief by saying:

*I decided to go to the hospital, and after several tests, I was told I had uterine cancer. Initially, I didn’t accept the diagnosis when the doctor informed me. But later, I had to accept it, though it was very difficult. I became very emotional and worried afterward about what would happen to me.* ***Woman B***

*[…] I went to the hospital, and after some tests, I was told I had endometrial cancer. When I heard the news, I didn’t believe it. I was shocked, I thought it was just something normal happening to me, not knowing it was cancer. For days, I was denying that it could be true.* ***Woman M***

#### Subtheme 6: Stressful experiences.

Participants expressed how the diagnosis and treatment created ongoing stress, with concerns about surgery, recovery, and the uncertainty of the future. Many described feeling emotionally drained and overwhelmed by the challenges they faced. They described this period as one of the most difficult times in their lives.

The journ*ey after I was informed about my condition was extremely stressful. I was constantly worried and overwhelmed, especially when I thought about the surgery and the uncertainty of what would happen to me. I could not sleep well, and my mind was always racing with fear.* ***Woman H***

Stress was often intensified by repeated hospital visits and exposure to other patients’ experiences, which heightened anxiety and fear.

*Each hospital visit increased my stress. Seeing other patients and hearing their stories made me more afraid.* ***Woman G***

For some participants, the prolonged nature of illness and physical decline compounded psychological distress, leading to feelings of despair and emotional breakdown.

*I cried a lot because I felt my life was over. The past two years have been very hard. I’ve been bedridden and weak most of the time. It is very stressful*. ***Woman C***

Others similarly described a sense of constant emotional strain and depletion:

*I was always thinking, always worrying. Even when nothing was happening, my heart was not at rest*. ***Woman D***

#### Subtheme 7: Shuttered aspirations.

Many participants, particularly those who were single, felt devastated by the potential loss of their ability to conceive. They described sadness, grief, and uncertainty about fulfilling their aspirations of motherhood, while some tried to mentally prepare for the possibility of infertility. They expressed their sorrow by saying:

*Even after treatment, they couldn’t guarantee that I would be able to get pregnant. Hearing this broke my heart because I realized I could not have the children I had always dreamed of having.* ***Woman A***

*The doctor said my womb was not removed, so I could still conceive, but at my age and after the surgery, I’m not sure if it’s possible. That thought sometimes makes me sad because I’ve always wished to be a mother.* ***Woman D***

*Before the surgery, the doctor explained that they would have to remove my uterus, and that meant I would not be able to give birth again. Hearing that was very painful for me because I had always hoped to have children someday.* ***Woman J***

It is worth noting that participants who already had more than one child were not particularly disturbed by the news that they would no longer be able to conceive. One participant remarked:

*The surgery and treatment affected my fertility, and I can no longer conceive. However, I accepted it peacefully because I already have children and felt content with that. Yes, I was told that the surgery would make me unable to give birth. I wasn’t worried or disturbed since I already had four children and was not planning to have more.* ***Woman G***

### Theme 2: Social challenges

Women diagnosed with gynecological cancer often experience profound social challenges that extend beyond their physical illness. Participants highlighted that their diagnosis and subsequent infertility had a significant impact on how they were perceived in their communities, affecting their relationships, social standing, and daily interactions. The social repercussions of the disease were compounded by a general lack of awareness about gynecological cancers, which led to misconceptions and judgmental attitudes.

#### Subtheme 1: Stigmatization.

Participants recounted being stigmatized by community members, colleagues, and friends who lacked understanding of their condition. In some cases, others falsely assumed they had HIV/AIDS or labeled them as barren simply because they could not conceive after treatment. This stigmatization caused participants to feel alienated, embarrassed, and emotionally vulnerable:

*People have been stigmatizing me, and some even say I’ve gotten HIV/AIDS. Some also say I can’t give birth again.* ***Woman C***

*I’ve been stigmatized by colleagues and friends in conversations. But usually, they don’t say it to me directly, but I know I’m the one they’re talking about. They don’t know I have this condition, but they know I don’t have children, so they think I’m barren.* ***Woman D***

#### Subtheme 2: Impaired sexual and intimate relationships.

The participants also discussed how the physical and physiological effects of gynecological cancer disrupted intimate relationships, particularly with their spouses. The condition affected their reproductive organs, causing symptoms such as bleeding, discharge, and pain that made sexual intimacy challenging or impossible. These changes not only caused physical discomfort but also emotional strain, as women felt unable to fulfill what they considered expected marital roles. They expressed their frustration by saying:

*Since I was diagnosed, I haven’t been intimate with my husband, which makes my husband worry sometimes.* ***Woman K***

*Before the condition, I was a married woman, and still I am. But due to the condition, I can’t fulfill all my obligations as a woman, there are times my husband will be in need of me as a woman, but due to my condition, there is nothing we can do*. ***Woman G***

### Theme 3: Coping strategies

This theme highlights strategies participants employed to cope with their diagnoses and the challenges associated with treatment. Participants demonstrated proactive engagement with their healthcare plans, emphasizing adherence to prescribed treatments, lifestyle modifications, and dietary changes. These coping steps reflect an understanding of the importance of self-management and personal responsibility in improving health outcomes and preventing complications.

#### Subtheme 1: Adherence to medication.

Participants expressed a strong commitment to following their prescribed treatment regimens, including taking medications on schedule and undergoing surgeries as recommended by healthcare professionals. This adherence was motivated by a desire to recover and regain control over their lives. They expressed this commitment by saying:

*This was a condition I knew nothing about. The most thing that helped me is that I paid attention to what the doctor said. I bought the medications they prescribed and took them as ordered. Also, the surgery I did helped me in a way that it gave me hope that I will be fine.* ***Woman B***

*Personally, I just focus on taking my medication regularly, as I was taught by the healthcare workers.* ***Woman A***

#### Subtheme 2: Dietary and lifestyle modifications.

Participants reported making intentional dietary changes to support their treatment, promote recovery, and prevent the progression of their condition. Many had adjusted their eating habits by reducing foods perceived to exacerbate their illness and increasing the consumption of nutritious, health-promoting foods.

*I used to take carbonated drinks, but after I was diagnosed with cancer, I did my own research, and I found out that the carbonated drinks are not good for someone with* *cancer. So, I’ve stopped consuming them totally. I also eat healthy nowadays with little consumption of carbohydrates and more of vegetables, fruits, and water*. ***Woman D***

*I also adjusted my lifestyle towards healthy eating, and doctors advised me against too much consumption of salt*. ***Woman B***

*As for me, I don’t eat anyhow; like, taking of these carbonated drinks or smoked fish is a big no for me. So, after the diagnosis I stuck strictly to a healthy diet. I added more vegetables to my food.* ***Woman E***

#### Subtheme 3: Faith-based acts.

All participants identified as Christians and emphasized turning to prayer and spiritual practices as a source of strength and healing. Faith served as both a coping mechanism and a source of emotional support, helping participants navigate the uncertainty and fear associated with their diagnosis. They expressed their faith by saying:

*I am a Christian, and I know God is the ultimate healer. So, I always prayed for divine healing because without God, I wouldn’t be alive*. ***Woman B***

*I prayed for mercy from God*. *Before the surgery, the pastor came to pray for me. God hears me anytime I pray; He is still healing me.* ***Woman H***

*I normally pray to God to strengthen me mentally and emotionally so that it doesn’t weigh me down, and I feel He has indeed strengthened me.* ***Woman F***

#### Subtheme 4: Psychological support.

Participants also drew strength from the encouragement and support of family members and close friends. This moral support helped them maintain a positive outlook and reinforced their motivation to adhere to treatments and pursue recovery. One participant explained:

*I also receive encouragement from my parents and siblings, and that has also helped me a lot in not dwelling on my condition, but I feel my mom has helped me the most in dealing with and accepting my condition. After my surgery the doctor also said I can adopt a child if I feel the need for one.* ***Woman F***

Another participant highlighted the support of a spouse and family members with healthcare knowledge:

*My husband encourages me a lot and tells me that things will get better with time. My sister’s daughter, who is a nurse, has supported me the most.* ***Woman G***

*[…] My parents comforted me and reminded me that life is more important and that I can still be a mother in other ways, such as adoption*. ***Woman J***

## Discussion

Gynecological cancers and their treatments profoundly disrupt fertility at a stage when reproductive potential is central to women’s identities, social positioning, and future life plans [1]. In exploring this impact among women of reproductive age at Ho Teaching Hospital, Ghana, this study revealed fertility loss as a complex and enduring consequence of cancer that extends beyond physiological impairment. Participants’ accounts illuminated deep social and psychological stressors embedded within gendered and cultural expectations of womanhood and motherhood. These experiences were further compounded by treatment-related morbidity, stigma surrounding reproductive cancers, financial strain, and disruptions to education, collectively intensifying emotional distress and shaping how women navigated diagnosis, treatment, and survivorship. Despite these challenges, participants articulated adaptive coping strategies grounded in treatment adherence, faith and spirituality, and social support, which fostered resilience and sustained hope. The findings underscore the need for holistic, culturally responsive care that integrates fertility counseling, psychosocial support, effective symptom management, and social protection for women living with gynecological cancers.

Participants consistently described fertility impairment as a profound disruption to self-concept, often articulated through feelings of incompleteness, diminished womanhood, and loss of purpose. This feeling is not merely biological but socially constructed, especially in contexts like Ghana, where motherhood is strongly tied to social acceptance and respect. Prior evidence emphasizes that infertility challenges women’s sense of self and belonging, often resulting in diminished self-worth [33]. Consistently, cancer survivors who experience infertility describe feelings of being “incomplete” or “broken,” highlighting the deep emotional toll of fertility loss [34,35].

Fear of rejection further intensified sources of distress, reflecting marital and gender norms that prioritize childbearing and position infertility as a relational risk [36]. Anticipated abandonment and social marginalization due to infertility undermined women’s self-worth and discouraged engagement in intimate relationships, amplifying emotional vulnerability. Similar findings were noted in a previous study where women with fertility issues feared that their partners may leave them for more “fertile” women [37]. Against this backdrop of social and relational insecurity, diagnosis and treatment decision-making were frequently described as terrifying moments marked by existential fear, uncertainty, and anticipatory grief, not only for threatened survival but also for foreclosed motherhood. These intersecting threats intensified cancer-related distress and shaped how women interpreted, navigated, and coped with treatment, particularly when survival was framed as coming at the expense of reproductive potential [17].

Stigmatization was a significant social stressor experienced by women living with gynecological cancers, arising from deeply rooted misconceptions that associate illness affecting reproductive organs with moral, sexual, or spiritual transgression [38]. Such stigma not only precipitated social isolation and judgment but also reinforced gendered expectations surrounding purity, fertility, and womanhood. These findings are consistent with previous studies reporting that stigma surrounding reproductive cancers remains a significant barrier to women seeking and accessing early healthcare for gynecological symptoms, often resulting in delayed diagnosis, limited support, and feelings of alienation and emotional distress [39]. In a similar vein, evidence showed that women with cervical or ovarian cancer often experienced feelings of embarrassment and self-blame, which hindered their willingness to seek help or disclose their condition [6]. The resulting avoidance and social isolation experienced by participants eroded social support networks, intensified psychological distress, and discouraged open disclosure and timely engagement with care, amplifying the overall burden of the disease beyond its physical manifestations.

The findings revealed that women’s coping strategies were deeply shaped by both medical knowledge and sociocultural values, illustrating adaptive efforts to regain control and sustain hope amid illness. Adherence to prescribed medications and recommended nutritional practices functioned as a treatment compliance and also as psychologically empowering actions that reinforced agency, trust in healthcare providers, and optimism about recovery [40–42]. Alongside these biomedical strategies, faith and spirituality emerged as central emotional resources, with prayer providing comfort, reducing anxiety, and offering existential meaning in the face of uncertainty [43,44]. Moral encouragement from family and social networks further strengthened resilience by mitigating isolation, enhancing emotional stability, and reinforcing adherence to treatment. Notably, the participants’ narratives also suggest that adoption was a socially recognizable and emotionally acceptable possibility within their support networks. For some women, family members and healthcare providers did not merely offer comfort but actively reframed motherhood beyond biological childbirth, presenting adoption as a viable path to fulfilling maternal identity. This is evident in accounts where mothers, spouses, and relatives reassured participants that they could still ‘be a mother in other ways.’ Such responses indicate a degree of familiarity and social acceptability surrounding adoption, which may have helped reduce distress linked to infertility and softened the perceived loss of motherhood.

### Limitations

Despite the important insights generated, this study has several limitations. First, the study relied on participants’ subjective accounts of their fertility experiences and treatment-related impacts, which may be influenced by recall difficulties, emotional distress, or social desirability, potentially shaping how women described sensitive issues such as infertility, stigma, and sexual functioning. Second, the study was conducted in a single tertiary referral facility (Ho Teaching Hospital), and participants were purposively recruited; therefore, the experiences captured may not fully reflect those of women receiving care in other regions of Ghana, lower-level facilities, distant rural communities, or women who disengaged from care. Third, the qualitative descriptive design enabled an in-depth understanding but did not allow quantification of the magnitude of fertility impairment or comparison of experiences by cancer type, treatment modality, or time since treatment, and causal inferences could not be made. Finally, because women who were critically ill or with mental health concerns were excluded, the study may under-represent the experiences of those with the most severe psychosocial burden.

## Conclusion

The study revealed that women whose fertility has been affected by gynecological cancers experience profound psychological, emotional, social, and economic challenges that deeply shape their quality of life. Feelings of incompleteness, fear of rejection, stigma, and financial strain were central to their lived realities. Despite these challenges, many women demonstrated resilience through faith, moral encouragement, adherence to medical advice, and openness to alternative pathways to motherhood, particularly adoption. These findings underscore the need for a holistic and culturally sensitive approach to care, one that recognizes the emotional and spiritual dimensions of healing alongside clinical management. Strengthening supportive care systems, improving public awareness, and enhancing the role of nurses in psychosocial interventions, including counseling on adoption and other family-building options, will be essential in promoting recovery, dignity, and hope among women living with gynecological cancers.

## Supporting information

S1 File Interview Guide. (DOCX)

## References

[1] Chitoran E, Rotaru V, Mitroiu M-N, Durdu C-E, Bohiltea R-E, Ionescu S-O, et al. Navigating fertility preservation options in gynecological cancers: a comprehensive review. Cancers (Basel). 2024;16(12):2214. doi: 10.3390/cancers16122214 38927920 PMC11201795

[2] Zhu B, Gu H, Mao Z, Beeraka NM, Zhao X, Anand MP, et al. Global burden of gynaecological cancers in 2022 and projections to 2050. J Glob Health. 2024;14:04155. doi: 10.7189/jogh.14.04155 39148469 PMC11327849

[3] Siegel RL, Giaquinto AN, Jemal A. Cancer statistics, 2024. CA: A Cancer Journal for Clinicians. 2024;74:12–49. doi: 10.3322/caac.21820 38230766

[4] Bray F, Laversanne M, Sung H, Ferlay J, Siegel RL, Soerjomataram I. Global cancer statistics 2022: GLOBOCAN estimates of incidence and mortality worldwide for 36 cancers in 185 countries. CA Cancer J Clin. 2024;74:229–63. doi: 10.3322/caac.21834 38572751

[5] Bruni L, Albero G, Serrano B, Mena M, Collado J, Gómez D, et al. Human Papillomavirus and Related Diseases in Ghana. 2023. https://hpvcentre.net/statistics/reports/GHA.pdf

[6] Binka C, Nyarko SH, Awusabo-Asare K, Doku DT. Barriers to the Uptake of Cervical Cancer Screening and Treatment among Rural Women in Ghana. Biomed Res Int. 2019;2019:6320938. doi: 10.1155/2019/6320938 31781631 PMC6874950

[7] World Health Organization. Cervical cancer Ghana 2021 country profile. https://www.who.int/publications/m/item/cervical-cancer-gha-country-profile-2021. 2021. Accessed 2026 January 20.

[8] Sefogah PE, Swarray-Deen A, Nsaful J, Attah DA, Moyer CA, Oppong SA. Breaking the Silence: Barriers to Fertility Preservation Among Young Breast Cancer Patients in Ghana - a Multicenter Study. Res Sq. 2025. doi: 10.21203/rs.3.rs-6649154/v1

[9] Bray F, Ferlay J, Soerjomataram I, Siegel RL, Torre LA, Jemal A. Global cancer statistics 2018: GLOBOCAN estimates of incidence and mortality worldwide for 36 cancers in 185 countries. CA Cancer J Clin. 2018;68:394–424. doi: 10.3322/caac.21492 30207593

[10] Stasenko M, Fillipova O, Tew WP. Fallopian Tube Carcinoma. J Oncol Pract. 2019;15(7):375–82. doi: 10.1200/JOP.18.00662 31283415

[11] Odhiambo M, Muteshi C. Bridging the gap: An outlook of oncofertility care in Africa. Int J Gynaecol Obstet. 2025;169(3):908–12. doi: 10.1002/ijgo.16175 39853745

[12] Di Nisio V, Daponte N, Messini C, Anifandis G, Antonouli S. Oncofertility and Fertility Preservation for Women with Gynecological Malignancies: Where Do We Stand Today?. Biomolecules. 2024;14(8):943. doi: 10.3390/biom14080943 39199331 PMC11353009

[13] Ojo AS, Lipscombe C, Araoye MO, Akinyemi O. Global uptake of fertility preservation by women undergoing cancer treatment: An unmet need in low to high-income countries. Cancer Epidemiol. 2022;79:102189. doi: 10.1016/j.canep.2022.102189 35605436

[14] Kuug AK, James S, Sihaam J-B. Exploring the cultural perspectives and implications of infertility among couples in the Talensi and Nabdam Districts of the upper east region of Ghana. Contracept Reprod Med. 2023;8(1):28. doi: 10.1186/s40834-023-00225-z 37076914 PMC10114423

[15] Tabong PT-N, Adongo PB. Understanding the social meaning of infertility and childbearing: a qualitative study of the perception of childbearing and childlessness in Northern Ghana. PLoS One. 2013;8(1):e54429. doi: 10.1371/journal.pone.0054429 23342158 PMC3546987

[16] Ekpor E, Brobbey SS, Kumah CY, Akyirem S. Experience of infertility-related stigma in Africa: a systematic review and mixed methods meta-synthesis. Int Health. 2025;17(6):903–13. doi: 10.1093/inthealth/ihaf060 40421541 PMC12585573

[17] Stroeken Y, Hendriks F, Beltman J, Ter Kuile M. Quality of Life and Psychological Distress Related to Fertility and Pregnancy in AYAs Treated for Gynecological Cancer: A Systematic Review. Cancers (Basel). 2024;16(20):3456. doi: 10.3390/cancers16203456 39456550 PMC11506014

[18] Yoshida K, Hashimoto T, Koizumi T, Suzuki N. Psychosocial experiences regarding potential fertility loss and pregnancy failure after treatment in cancer survivors of reproductive age to identify psychosocial care needs: a systematic review. Support Care Cancer. 2024;32(6):337. doi: 10.1007/s00520-024-08544-w 38727728

[19] Stal J, Benedict C, Partridge AH, Pozo-Kaderman C, Mack JW. Fertility preservation and mental health among cancer patients of reproductive age. Fertil Steril. 2025;124(4):612–8. doi: 10.1016/j.fertnstert.2025.07.007 40764167 PMC12360784

[20] He X-Y, Huang Y, Qiao C-P, Ma J, Han X, Fan X-M, et al. Fertility anxiety partially mediates depression and recurrence fear in reproductive-age cervical cancer patients: A cross-sectional study. World J Clin Oncol. 2025;16(9):110031. doi: 10.5306/wjco.v16.i9.110031 41024840 PMC12476602

[21] Ouh Y-T, Kim E-Y, Kim NK, Lee N-W, Min K-J. Impact of Depression and/or Anxiety on Mortality in Women with Gynecologic Cancers: A Nationwide Retrospective Cohort Study. Healthcare (Basel). 2025;13(15):1904. doi: 10.3390/healthcare13151904 40805936 PMC12345817

[22] Sefogah PE, Attah DA, Swarray-Deen A, Nsaful J, Oppong SA, Moyer CA. Perspectives on fertility preservation among women living with breast cancer in Ghana. BMC Womens Health. 2025;25(1):409. doi: 10.1186/s12905-025-03963-1 40859257 PMC12382290

[23] Sandelowski M. Whatever happened to qualitative description?. Research in Nursing & Health. 2000;23(4):334–40. doi: 10.1002/1098-240X(200008)23:4<334::AID-NUR9>3.0.CO;2-G 10940958

[24] Sandelowski M. What’s in a name? Qualitative description revisited. Res Nurs Health. 2010;33(1):77–84. doi: 10.1002/nur.20362 20014004

[25] Creswell JW, Creswell DJ. Research design: Qualitative, quantitative, and mixed methods approaches. 5th ed. Thousand Oaks, CA, US: SAGE Publications Inc. 2017.

[26] Tong A, Sainsbury P, Craig J. Consolidated criteria for reporting qualitative research (COREQ): a 32-item checklist for interviews and focus groups. Int J Qual Health Care. 2007;19(6):349–57. doi: 10.1093/intqhc/mzm042 17872937

[27] Vasileiou K, Barnett J, Thorpe S, Young T. Characterising and justifying sample size sufficiency in interview-based studies: systematic analysis of qualitative health research over a 15-year period. BMC Med Res Methodol. 2018;18(1):148. doi: 10.1186/s12874-018-0594-7 30463515 PMC6249736

[28] Kallio H, Pietilä A-M, Johnson M, Kangasniemi M. Systematic methodological review: developing a framework for a qualitative semi-structured interview guide. J Adv Nurs. 2016;72(12):2954–65. doi: 10.1111/jan.13031 27221824

[29] Saunders B, Sim J, Kingstone T, Baker S, Waterfield J, Bartlam B, et al. Saturation in qualitative research: exploring its conceptualization and operationalization. Qual Quant. 2018;52(4):1893–907. doi: 10.1007/s11135-017-0574-8 29937585 PMC5993836

[30] Braun V, Clarke V. Using thematic analysis in psychology. Qualitative Research in Psychology. 2006;3(2):77–101. doi: 10.1191/1478088706qp063oa

[31] Kumar S, Barolia DR, Petrucka DP, Ali MAA. RIGOR: THE ASSESSMENT OF TRUSTWORTHINESS. Kashf Journal of Multidisciplinary Research. 2025;2:10–9. doi: 10.71146/kjmr204

[32] World Medical Association. WMA Declaration of Helsinki – Ethical Principles for Medical Research Involving Human Participants. https://www.wma.net/policies-post/wma-declaration-of-helsinki/. 2016. Accessed 2026 May 22.

[33] Akintayo AA, Aduloju OP, Dada MU, Abiodun-Ojo OA, Oluwole LO, Ade-Ojo IP. Comparison of self-esteem and depression among fertile and infertile women in a low resource setting. J Obstet Gynaecol. 2022;42(5):1198–203. doi: 10.1080/01443615.2021.1945002 34379544

[34] Barbosa C, Santos S, Pedro J. Infertility-related stress and quality of life: the role of experiential avoidance. Current Psychology. 2025;44:17243–52. doi: 10.1007/s12144-025-08379-6

[35] Sira N, McNeil S, Hegde A, Geistman K, Schwartz A. Infertility and Identity: A Closer Look Into Experiences of Emerging Young Adult Childhood Cancer Survivors. J Pediatr Hematol Oncol Nurs. 2024;41(1):32–43. doi: 10.1177/27527530231190386 37858932

[36] Ofosu-Budu D, Hanninen V. Living as an infertile woman: the case of southern and northern Ghana. Reprod Health. 2020;17(1):69. doi: 10.1186/s12978-020-00920-z 32434580 PMC7240982

[37] Boivin J, Carrier J, Zulu JM, Edwards D. A rapid scoping review of fear of infertility in Africa. Reprod Health. 2020;17(1):142. doi: 10.1186/s12978-020-00973-0 32928239 PMC7488744

[38] Asakitogum DA, Aziato L, Ohene LA. Ghanaian women beliefs on the causes, prevention and treatment of cervical cancer: A qualitative Study. Int J Africa Nursing Sci. 2023;18:100538. doi: 10.1016/j.ijans.2023.100538

[39] Tuck CZ, Cooper R, Aryeetey R, Gray LA, Akparibo R. A critical review and analysis of the context, current burden, and application of policy to improve cancer equity in Ghana. Int J Equity Health. 2023;22(1):254. doi: 10.1186/s12939-023-02067-2 38066530 PMC10709985

[40] Achieng C, Bunani N, Kagaayi J, Nuwaha F. Adherence to antiretroviral and cancer chemotherapy, and associated factors among patients with HIV-cancer co-morbidity at the Uganda Cancer Institute: a cross sectional study. BMC Public Health. 2023;23(1):1451. doi: 10.1186/s12889-023-16387-z 37507710 PMC10386774

[41] Okyere Asante PG, Owusu AY, Oppong JR, Amegah KE, Nketiah-Amponsah E. An assessment of the direct and indirect costs of breast cancer treatment in leading cancer hospitals in Ghana. PLoS One. 2024;19(5):e0301378. doi: 10.1371/journal.pone.0301378 38771827 PMC11108162

[42] de Vries-Ten Have J, Winkels RM, Kampman E, Winkens LHH. Behaviour change techniques used in lifestyle interventions that aim to reduce cancer-related fatigue in cancer survivors: a systematic review. Int J Behav Nutr Phys Act. 2023;20(1):126. doi: 10.1186/s12966-023-01524-z 37833784 PMC10576285

[43] Kugbey N, Oppong Asante K, Meyer-Weitz A. Illness perception and coping among women living with breast cancer in Ghana: an exploratory qualitative study. BMJ Open. 2020;10(7):e033019. doi: 10.1136/bmjopen-2019-033019 32665380 PMC7365420

[44] Afaya A, Anaba EA, Bam V, Afaya RA, Yahaya AR, Seidu AA, et al. Socio-cultural beliefs and perceptions influencing diagnosis and treatment of breast cancer among women in Ghana: a systematic review. BMC Women’s Health. 2024;24:288. doi: 10.1186/s12905-024-03106-y 38745160 PMC11092234

