# Supplementary material for: Fertility in the Shadow of Cancer: Experiences of Reproductive Loss Among Women with Gynecological Cancers in Ghana
Source: PLoS One. 2026 Aug 14;21(8):e0343859. doi: 10.1371/journal.pone.0343859 (PMC13475906; doi:10.1371/journal.pone.0343859)
Supplement: Supplementary file 1 [file pone.0343859.s001.docx]

**S1 File. Interview Guide**

**A: Background Information**

1. Can you please tell me a little about yourself?
   a) Age
   b) Marital Status
   c) Number Of Children
   d) Occupation
   e) Educational Level
   f) Religion
   g) Type Of Cancer
   h) Ethnicity

**B: Perceptions and Understanding of Gynecological Cancers and Fertility**

1. What do you know about gynecological cancers?
   a) Probe: How did you come to learn about it? (e.g., media, health professionals, family/friends)
2. What is your understanding of how gynecological cancers may affect fertility?
   a) Probe: Do you think it can influence a woman’s ability to have children? How?
3. What concerns, if any, do you have about fertility and gynecological cancers?

**C: Challenges Faced by Women with Gynecological Cancers**

1. Since your diagnosis, what challenges have you faced in your day-to-day life?
   a) Probe: Physical challenges (pain, treatment side effects, infertility, etc.)
   b) Probe: Emotional/psychological challenges (stress, anxiety, stigma, relationships)
   c) Probe: Financial/social challenges (cost of treatment, support from family/community)
2. How have these challenges affected your family life, work, or social relationships?
3. Are there specific cultural or community-related challenges you have experienced?
4. Have you experienced any form of stigma or discrimination because of your condition?
   a) Probe: From family members (changes in how they relate to you)
   b) Probe: From friends or colleagues (exclusion, labeling, gossip, reduced support)
   c) Probe: From the wider community (social isolation, negative cultural beliefs, misconceptions about cancer)

**D: Coping Strategies**

1. How have you been coping with the challenges you just described?
   a) Probe: What practical steps do you take (e.g., lifestyle adjustments, seeking medical support)?
   b) Probe: What emotional or psychological strategies do you use (e.g., prayer, counseling, support groups)?
2. Who or what has supported you most in dealing with your condition?
   a) Probe: Family, friends, religious groups, health professionals, community networks.
3. What advice would you give to other women diagnosed with gynecological cancers about coping with challenges?

**E: Conclusion**

1. Is there anything else you would like to share about your experience with gynecological cancer that we have not discussed?
2. What do you think health professionals or policymakers could do to better support women living with gynecological cancers?

Thank you
